# Supplementary material for: DPP-IV Inhibitory Peptides GPF, IGL, and GGGW Obtained from Chicken Blood Hydrolysates
Source: Int J Mol Sci. 2022 Nov 16;23(22):14140. doi: 10.3390/ijms232214140 (PMC9696969; doi:10.3390/ijms232214140)
Supplement: Supplementary file 1 [file ijms-23-14140-s001.zip › ijms-1967992-supplementary.pdf]

Supplementary data Table S1. Sequences of the peptides identified in fraction 2 obtained after RP-HPLC separation of AP and APP chicken blood hydrolysates. Identification was performed by mass spectrometry in tandem.

	AP SAMPLE						
**Num**	**Names**	**Sequence**	**Obs MW[a]**	**Obs m/z[b]**	**Theor MW[c]**	**Theor m/z[d]**	**Theor z[e]**
1	Uncharacterized protein	VAEQELLDASERVQL	1698.89	567.30	1698.88	567.30	3
2	Uncharacterized protein	VAEQELLDASERVQLL	1811.97	906.99	1811.96	906.99	2
3	Uncharacterized protein	LLDASERVQL	1142.63	572.32	1142.63	572.32	2
4	Histone H3	IAQDFKTDLRFQ	1480.77	494.60	1480.77	494.60	3
5	6-phosphogluconate dehydrogenase, decarboxylating	GLLFVGSGVSGGEEGAR	1590.81	796.41	1590.80	796.41	2
6	Collagen alpha-1(I) chain	FSFLPQPPQEKAHDGGRYY	2236.08	560.03	2236.07	560.02	4
7	Collagen alpha-1(I) chain	FLPQPPQEKAHDGGRYY	2001.98	501.50	2001.97	501.50	4
8	Actin, aortic smooth muscle	LAGRDLTDY	1022.50	512.26	1022.50	512.26	2
9	Actin, aortic smooth muscle	NVPIYEGY	953.45	477.73	953.45	477.73	2
10	Actin, aortic smooth muscle	VWIGGSILASL	1114.64	558.33	1114.64	558.33	2
11	Actin, aortic smooth muscle	IIAPPERKYS	1172.66	391.89	1172.65	391.89	3
12	Actin, aortic smooth muscle	FIGMESAGIH	1076.50	539.26	1076.50	539.25	2
13	Uncharacterized protein	IVAGAMVVIVVIFV	1428.76	715.39	1428.88	715.45	2
14	REVERSED C1q domain-containing protein	PVGPMGPLGPA	991.54	496.77	991.52	496.77	2
15	SERPIN domain-containing protein	QAVNFKTDAEQARAQIN	1885.96	629.66	1885.93	629.65	3
16	REVERSED Elastin	GPVGPVGAVG	808.46	405.24	808.44	405.23	2
17	REVERSED VWFA domain-containing protein	GTEGPLGPLGPQ	1121.55	561.78	1121.57	561.79	2
18	REVERSED VWFA domain-containing protein	GEKGPLGPNGPVGV	1276.81	639.41	1276.68	639.35	2
19	REVERSED VWFA domain-containing protein	RDGPQGPLGPAG	1121.55	561.78	1121.54	561.78	2
20	Collagen IV NC1 domain-containing protein	LPGIPGQDGPPGPPGIPGCNGTK	2124.99	709.34	2125.06	709.36	3
21	Alpha 1 type IIA collagen	GPQGKVGPTGAPG	1122.55	562.28	1122.57	562.29	2
22	Alpha 1 type IIA collagen	GPAGAPGFPGAPGSKGEAGPTGARG	2146.09	537.53	2146.05	537.52	4
23	RIX1 domain-containing protein	LGLRHQAGVEPGGAGAA	1559.81	520.94	1559.82	520.95	3

24	SERPIN domain-containing protein	EMISMLIALPTENT	1561.79	781.90	1561.77	781.89	2
25	Uncharacterized protein	AVASSGMSKMLPSVP	1460.71	731.36	1460.74	731.38	2
26	Chloride channel protein	AAAAGVSVAFGAP	1087.50	544.76	1087.57	544.79	2
27	MFS domain-containing protein	GLRDLLGTEDMWNVLLSFSG	2222.05	556.52	2222.10	556.53	4
28	Hemoglobin subunit alpha-A	NHIDDIAGT	954.44	478.23	954.44	478.22	2
29	Glucosamine-6-phosphate isomerase	SSRTRLKTLAMDTILANAK	2089.10	697.37	2089.17	697.40	3
30	ATP-dependent 6-phosphofructokinase	AGLLDELLRDGLISE	1612.85	807.43	1612.87	807.44	2
31	REVERSED Uncharacterized protein	TSIRSAQIAVLNGGVGNI	1769.02	590.68	1768.98	590.67	3
32	REVERSED HECT domain-containing protein	SLASTGGFLSRTASSGSGGPPP	1989.99	664.34	1989.97	664.33	3
33	REVERSED Uncharacterized protein	GAGLLLLEALEKGYWV	1730.98	578.00	1730.96	577.99	3
34	REVERSED Uncharacterized protein	LKATAPVRN	968.63	485.32	968.58	485.30	2
35	REVERSED LAM_G_DOMAIN domain-containing protein	GPAGDAGAEGKPGIPG	1349.67	450.90	1349.66	450.89	3
36	Transient receptor potential ankyrin 1	LRSFIKKN	1004.72	503.37	1004.61	503.31	2
37	Beta-2-microglobulin (Fragment)	NCFAAGF	728.36	365.19	728.30	365.15	2
38	TYR_PHOSPHATASE_2 domain-containing protein	VAGLGRAPVLVAL	1234.87	618.44	1234.78	618.39	2
39	Uncharacterized protein	LGYNDYVK	970.46	486.24	970.48	486.24	2
40	Uncharacterized protein	ELSDQLKAKN	1144.69	573.35	1144.61	573.31	2
41	Uncharacterized protein	RKSGESANAVP	1114.64	558.33	1114.57	558.29	2
42	REVERSED Threonine synthase-like 2	LLPICSPMFLGGDPAYGSFLA	2168.15	723.72	2168.07	723.70	3
43	REVERSED Collagen alpha-1(VIII) chain	GMPGIEGKP	884.43	443.22	884.44	443.23	2
44	Uncharacterized protein	APESLLAGQN	998.45	500.23	998.50	500.26	2
45	REVERSED Uncharacterized protein	ARSSKTTGRQVDKT	1533.77	767.89	1533.82	767.92	2
46	REVERSED Uncharacterized protein	LGPFSFSFGPAT	1226.58	614.30	1226.60	614.30	2
47	REVERSED RIX1 domain-containing protein	EGWGGRGGGYGRASRT	1622.79	812.40	1622.76	812.39	2
48	REVERSED Uncharacterized protein	PGPAASVREALVAPQLGGAAP	1928.02	643.68	1928.05	643.69	3
49	Uncharacterized protein	LPQGCKAAAPQRARSVPAV	1919.01	960.51	1919.05	960.53	2
50	Fanconi anemia group C protein homolog	SLLDQLTSLQINSLEK	1800.93	601.32	1800.98	601.33	3
51	RanBP2-type domain-containing protein	ALLRGEGR	870.63	436.32	870.50	436.25	2

52	REVERSED XRCC1_N domain-containing protein	RQRSGPQRRARKPA	1662.82	555.28	1662.96	555.33	3	
53	REVERSED Uncharacterized protein	GNGGGWGNSGGGGGGGGGGNGDGGGGDGCGNSGGCGGGGG	2923.76	585.76	2924.02	585.81	5	
54	Elongation factor 1-alpha	IGGIGTVPVGR	1024.60	513.31	1024.60	513.31	2	
55	REVERSED ULP_PROTEASE domain-containing protein	SPSKRFRGWRARTERT	1989.99	664.34	1990.07	664.36	3	
56	Ovocleidin-17	PTWALLGCVLLLPSLR	1751.19	584.74	1751.01	584.68	3	
57	Protein kinase domain-containing protein	LTEVTTCYSVCHHEVLG	1889.96	630.99	1889.86	630.96	3	
58	Uncharacterized protein	PMADSGCLTEGEMGLIFVN	1982.82	661.95	1982.88	661.97	3	
59	Uncharacterized protein	GAIEILLTYIVPQAAI	1683.98	843.00	1683.98	843.00	2	
60	Uncharacterized protein	RAAELRPLR	1080.74	541.38	1080.65	541.33	2	
61	REVERSED CDH3	DDDADTANVRMVS	1407.70	704.86	1407.59	704.80	2	
62	REVERSED Uncharacterized protein	ELEAAEEQQARWQLV	1798.90	600.64	1798.88	600.63	3	
63	Non-specific serine/threonine protein kinase	VGTADGTLAVFEDR	1449.79	725.90	1449.71	725.86	2	
64	Non-specific serine/threonine protein kinase	GEGPPRVDFVD	1186.54	594.28	1186.56	594.29	2	
65	REVERSED DNA helicase; REVERSED Bloom syndrome protein homolog	LIKDLVLK	940.62	471.312	940.63	471.32	2	
66	Ribonuclease H	GKQYVLVGVEIISGLLQ	1814.98	606.00	1815.05	606.02	3	
67	Uncharacterized protein	AQQPVQSELVQRCQ	1612.84	538.62	1612.80	538.61	3	
68	VWFD domain-containing protein; Uncharacterized protein	DFSLHVSYDAD	1267.52	634.77	1267.53	634.77	2	
69	Serine/threonine-protein kinase 10	RELVAEAKAE	1114.64	558.33	1114.60	558.31	2	
70	REVERSED ATP-grasp domain-containing protein; REVERSED Succinate--CoA ligase [ADP-forming] subunit beta, mitochondrial	KAIKYAEE	950.52	476.26	950.51	476.26	2	
71	REVERSED DUF676 domain-containing protein	LLMKIKFI	1004.72	503.37	1004.64	503.33	2	
72	REVERSED Integrator complex subunit 2	PLEAQLICLVDAAEE	1612.84	807.43	1612.80	807.41	2	
73	REVERSED Uncharacterized protein	CVLKIRGV	886.65	444.33	886.54	444.28	2	
74	REVERSED Fanconi_A_N domain-containing protein; REVERSED Fanconi anemia protein FancA	KTVANKAALVATP	1282.90	642.46	1282.76	642.39	2	
75	REVERSED Uncharacterized protein; REVERSED Inhibitor_I29 domain-containing protein	PLQFCKRANVEK	1431.73	716.87	1431.76	716.89	2	
76	REVERSED Sodium/hydrogen exchanger	FIGLFIGISKFMAT	1543.82	515.61	1543.85	515.62	3	
77	REVERSED Uncharacterized protein; REVERSED Monocarboxylate transporter 10	AWIRYSKVKFVSFNF	1891.09	631.37	1891.01	631.34	3	
78	REVERSED SAM domain-containing protein; REVERSED SAM domain-containing	GNGRNRCGDPIEKT	1515.79	758.90	1515.72	758.87	2	

79	REVERSED Protein-tyrosine-phosphatase	YVIDKPPDGPAGESTRFI	1960.98	654.67	1960.99	654.67	3
	**APP SAMPLE**						
	**Names**	**Sequence**	**Obs MW[a]**	**Obs m/z[b]**	**Theor MW[c]**	**Theor m/z[d]**	**Theor z[e]**
80	Hemoglobin subunit alpha-A	TTYPPTKTYFPH	1451.71	484.91	1451.71	484.91	3
81	Hemoglobin subunit alpha-A	NHIDDIAGT	954.44	478.23	954.44	478.23	2
82	Hemoglobin subunit alpha-D	LSPGSDQVRGH	1151.57	384.86	1151.57	384.86	3
83	REVERSED DUF4550 domain-containing protein	KCYTPVCLK	1053.55	352.19	1053.53	352.18	3
84	Uncharacterized protein	FITSAFLT	898.48	450.25	898.48	450.23	2
85	Citrate synthase	VVPGYGHAVLR	1166.66	389.89	1166.65	389.89	3
86	Transcriptional adapter	QARALIKIDVNK	1367.81	684.91	1367.82	684.92	2
87	Collagen-alpha-3 type IX	VGLPGPIGAP	876.44	439.23	876.51	439.26	2
88	REVERSED Uncharacterized protein	TEKVMEDSPLRYLAL	1763.89	588.97	1763.91	588.98	3
89	REVERSED VWFA domain-containing protein	GTEGPLGPLGPQ	1121.55	561.78	1121.57	561.79	2
90	REVERSED Helicase ATP-binding domain-containing protein	AAELDQVEKVLRQ	1497.80	500.27	1497.81	500.28	3
91	REVERSED Ovocleidin-116	VTSPASAVEPQT	1185.58	593.80	1185.59	593.80	2

a: Molecular weight observed in the Q-TOF mass spectrometer.

b: Mass/charge ratio observed in the Q-TOF mass spectrometer.

c: Theoretical molecular weight calculated from the identified peptide sequence.

d: Theoretical mass/charge ratio calculated from the identified peptide sequence.

e: Theoretical charge of identified peptide sequence in the Q-TOF mass spectrometer.
